# Relapsed Symptoms in a Patient Treated for Pubic Symphysis Septic Arthritis/Adjacent Osteomyelitis: Diagnostic Challenges

**DOI:** 10.1155/2020/8819268

**Published:** 2020-11-26

**Authors:** Shankar Upadhyayula

**Affiliations:** Division of Pediatric Infectious Diseases, Akron Childrens Hospital, Akron, OH, USA

## Abstract

We describe the case of a 14-year-old girl with relapsed pain following adequate treatment of pubic symphysis septic arthritis with adjacent osteomyelitis. Evaluation of her symptoms was challenging, because magnetic resonance imaging (MRI) was not helpful and repeat surgical exploration was not favored. She was treated with a combination of prolonged antimicrobial therapy and local steroid injection. This case highlights the limitations of MRI as a follow-up study for evaluating symptom relapse of acute osteomyelitis.

## 1. Introduction

Septic arthritis of the pubic symphysis with adjacent osteomyelitis is rare and accounts for less than 1% of all anatomical sites with reported osteomyelitis [1, 2]. Presenting symptoms include fever, pubic/groin pain, and antalgic gait. Diagnosis is often delayed due to the low index of suspicion [3–9]. Most common organisms isolated are *Staphylococcus aureus* and *Pseudomonas aeruginosa* followed by polymicrobial infections [3]. Sports especially soccer and others that require forceful hip adduction, female incontinence surgery, pelvic malignancy, and IV drug use are risk factors [3–9]. Osteitis pubis is an inflammatory condition that remains in the differential and is distinguished by lack of positive cultures from the site of infection [7].

We describe the case of a young girl with pubic symphysis septic arthritis/osteomyelitis who relapsed with worsening pain after treatment. To the best of our knowledge, none of the previously published reports address the challenges related to evaluating relapsed symptoms in this patient population.

## 2. Case Description

A 14-year-old, previously healthy Caucasian girl, developed right lower quadrant and suprapubic abdominal pain. She had nausea but no diarrhea or urinary symptoms. Initial visit to her pediatrician led to the diagnosis of muscular pain, and her numerical pain rating scale (NPRS) was 4 (range 0–10). The patient recounted having pulled a muscle in her groin when she was on track. She was advised to use nonsteroidal anti-inflammatory medication. Pain progressively worsened over several days including difficulty falling asleep, and a day prior to admission, she developed fevers. She was brought to the emergency room (ER). Records from the ER showed a temperature of 39.1 degrees Celsius, NPRS-9. Other vitals were stable. Examination revealed pain in the right lower quadrant and suprapubic area with tenderness at McBurney point. Ultrasound scan of the abdomen was not able to visualize the appendix, and uterus and adnexa were normal. Urine analysis with microscopy was normal. Urine pregnancy test was negative. Laboratory testing revealed white blood count of 7.9 10E9/L with 12% bands and 66% neutrophils. C-reactive protein (CRP) was elevated to 29.8 mg/dl (normal range, 0.0–1.0 mg/dL). Metabolic panel was within the normal limits. Due to suspicion for appendicitis, a computerized tomographic (CT) scan of abdomen and pelvis was performed and was remarkable for nonspecific fluid in the pelvis and edema in the extraperitoneal space of the pelvis suspicious for cystitis, with a difficult to identify appendix along with air fluid levels in the small bowel suspicious for gastroenteritis. General surgery was consulted, but felt no surgical intervention was necessary. Subsequently, she was admitted to the hospital.

Over the next 48 hours, she continued to require frequent doses of pain medication and had ongoing fevers. Repeat CRP remained elevated at 30.3 mg/dl. She underwent magnetic resonance enterography (MRE) with a suspicion for inflammatory bowel disease. MRE demonstrated septic arthritis of the pubic symphysis with adjacent phlegmon/abscess and probable associated osteomyelitis. She was taken to the operating room, and intraoperative findings were notable for large amount of pus under the periosteum. Cultures grew methicillin-susceptible *staphylococcus aureus*. She was placed on IV cefazolin, and by the 3^rd^ postoperative day, CRP was 13.5 mg/dl (more than 50% improved). She was discharged on oral cephalexin at 1250 mg three times daily. After completing a 4-week course of antibiotic therapy, CRP was normal at 0.5 mg/dl. Functionally, she was much improved but complained of discomfort in her left groin area and the left upper thigh up on waking up in the morning, after long walks, etc, NPRS-2. This was attributed to stiffness and deconditioning. Antibiotics were discontinued.

**Figure 1:**
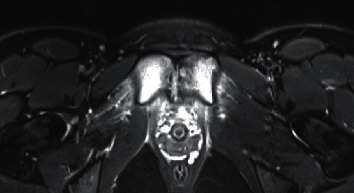
MR image when the patient relapsed with pain.

**Figure 2:**
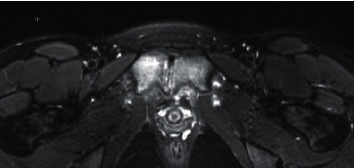
MRI after completion of therapy (about 6 months later).

Patient returned to the infectious disease clinic a month later with significant worsening of left groin pain, NPRS-8. Unlike her last clinic visit, she now had pain most of the time (not only on waking up or after exertion). She had no systemic symptoms, and complete blood count and CRP were within normal limits. Repeat MRI was obtained, and it showed progression of destructive changes at the pubic symphysis with probable small residual abscess posterior to the pubic symphysis. No change in bone marrow edema in both ischiopubic rami (Figure 1). Following consultation with orthopedic surgery, a percutaneously inserted central catheter (PICC) was placed and she was treated with intravenous cefazolin. She did not show satisfactory improvement in symptoms after 6 weeks of antibiotic therapy and underwent CT-guided aspiration of the small residual abscess. Aspirate was sent for culture and broad range polymerase chain reaction for bacteria and fungus. All infectious testing resulted negative. Local injection of triamcinolone followed. She started feeling better about a few days later and completed a total of 5 months of antibiotic therapy (6 weeks of IV plus 14 weeks of oral antibiotics). By this time, she was much improved clinically and denied pain NPRS-0. Repeat MRI was performed a few weeks after stopping antibiotics and showed edema and enhancement of the superior and inferior pubic rami which were similar in appearance to the previous study. Decreased fluid was observed in the pubic symphysis. Slightly increased edema and fluid in the soft tissues were observed between pubic symphysis and bladder (Figure 2). At the time of writing this report, she was 11 months out from her last MRI and is doing extremely well.

## 3. Discussion

Our patient was a previously healthy young athlete who developed right lower quadrant/suprapubic pain and fever. She had delayed diagnosis but responded well to a combination of surgical drainage and antibiotic therapy. Unfortunately, she relapsed a month later with worsening pain. Evaluating the cause of her relapse was challenging. She was systemically well and had normal labs. MRI showed progression of bone changes with probable small residual abscess. The difficulty was distinguishing recrudescent infection from sterile inflammation.

One approach would have been to take her back to the operating room for debridement and culture, but orthopedic surgery was worried about scarring in a vital area and wanted to manage her conservatively. She was treated with intravenous antibiotics, and when symptoms did not improve after 6 weeks, interventional radiology-guided aspiration was performed. No pus could be aspirated; only tissue fluid was obtained. Infectious evaluation was negative. Due to the possibility that she had ongoing sterile inflammation, she was given intralesional triamcinolone.

Literature review of septic arthritis of pubic symphysis with adjacent osteomyelitis in children revealed the following [3–9].

Ross and Hu. [3] reviewed 100 cases of septic arthritis of pubic symphysis. Most of their patients were adults with a mean age of 48 years (range 7–86). Common reported symptoms included pubic pain (68%), fever (74%), and painful gait (59%). Bacteremia was noted in 73%. In this series, 3 patients relapsed. 1 of them responded to antibiotic therapy, and 2 needed incision and drainage along with antibiotics.

Tsai and Chen [4] report the case of a previously heathy 8-year-old boy with thigh pain for almost two weeks followed by fever and systemic inflammation. Blood cultures were negative. MRI confirmed the diagnosis, and he responded well to medical management.

Hartshorn et al. [5] reported a case of a 11-year-old who was a soccer player. He had bilateral groin pain for 4 weeks and antalgic gait for 2 weeks before the diagnosis was made. MRI confirmed the diagnosis, but no culture data were available. The patient responded to 6 weeks of antimicrobial therapy.

Lorenzo et al. [6] reported the case of a 12-year-old ballet dancer who had a 2-week history of groin pain followed by fever and malaise. Pelvic CT showed an extraperitoneal abscess, and methicillin-susceptible *staphylococcus aureus* was recovered from the abscess. She responded to 4 weeks of cloxacillin therapy.

Alqahtani et al. [5] report a case of symphysis pubis osteomyelitis that was misdiagnosed and progressed to bilateral adductor abscesses. Their patient was a 17-year-old with groin pain for 3 weeks and fever and chills for 2 weeks before an MRI diagnosed the problem. Operative room cultures identified group A streptococcus. Patient completed a total of 6 weeks of antibiotics and made full recovery.

Jasmijn et al. [8] and Nitsche et al. [9] report pubic symphysis septic arthritis in two previously healthy 16- and 13-year olds, respectively. *Staphylococcus aureus* was isolated in both of these patients. They responded well to a combination of drainage and antimicrobial therapy.

Data on managing relapsed symptoms in patients with pubic symphysis septic arthritis/osteomyelitis is extremely limited. Clinicians often resort to imaging to evaluate the relapse. However, MRI as a follow-up study for acute osteomyelitis has limitations [10–15].

Visconti et al. [10] report that follow-up imaging among vertebral osteomyelitis cases with clear clinical improvement more likely shows stability or worsening bony changes rather than improvement.

Warmann et al. [11] report that both MRI and scintigraphy can reveal persistent and even additional positive signal changes at the site of infection or other locations nearby when used as follow-up studies. It is difficult to determine if these findings are reparative or are inflammatory in origin. More so, these signals can possibly be observed over a long period, during which patients may have normal serum parameters and no/nonspecific clinical symptoms. They claim that positron-emission tomography (PET)/CT may be superior to MRI in differentiating repair from ongoing inflammation. Lee et al. [12] report fluorodeoxyglucose PET as the most sensitive radionuclide modality for evaluating chronic osteomyelitis.

Gilliams et al. [13], Zarrouk et al. [14], and Baxi et al. [15] report that imaging abnormalities on MRI can persist despite clinical improvement. Routine follow-up MRI did not seem to correlate with clinical follow-up among patients with spinal infections.

Our patient likely improved with a combination of prolonged antibiotic therapy and local steroid injection. Of note, her MRI findings several weeks after she was off antibiotic therapy were essentially unchanged from prior appearances. At the time of this report, 12 months out from completion of antimicrobial therapy, she is pain free, NPRS-0, and is functionally back to preillness baseline. Repeat MRI scan was suggested but was not pursued due to patient preference related to the cost of the study.

## 4. Conclusion

Our case highlights that MRI scans are likely to remain unchanged/worsen when used as a follow-up study for acute osteomyelitis. This can be particularly challenging when patients present with relapsed symptoms and surgical access to the involved site is difficult. There is a need to evaluate other modalities such as PET/CT in this setting.

## Data Availability

No data were used to support this study.
